# Iron Flocs and the Three Domains: Microbial Interactions in Freshwater Iron Mats

**DOI:** 10.1128/mBio.02720-20

**Published:** 2020-12-15

**Authors:** Chequita N. Brooks, Erin K. Field

**Affiliations:** a Department of Biology, East Carolina University, Greenville, North Carolina, USA; University of Texas Health Science Center at Houston

**Keywords:** freshwater, iron mat, iron-oxidizing bacteria, microbial ecology, microbial interactions

## Abstract

Freshwater iron mats are dynamic geochemical environments with broad ecological diversity, primarily formed by the iron-oxidizing bacteria. The community features functional groups involved in biogeochemical cycles for iron, sulfur, carbon, and nitrogen. Despite this complexity, iron mat communities provide an excellent model system for exploring microbial ecological interactions and ecological theories *in situ*. Syntrophies and competition between the functional groups in iron mats, how they connect cycles, and the maintenance of these communities by taxons outside bacteria (the eukaryota, archaea, and viruses) have been largely unstudied. Here, we review what is currently known about freshwater iron mat communities, the taxa that reside there, and the interactions between these organisms, and we propose ways in which future studies may uncover exciting new discoveries. For example, the archaea in these mats may play a greater role than previously thought as they are diverse and widespread in iron mats based on 16S rRNA genes and include methanogenic taxa. Studies with a holistic view of the iron mat community members focusing on their diverse interactions will expand our understanding of community functions, such as those involved in pollution removal. To begin addressing questions regarding the fundamental interactions and to identify the conditions in which they occur, more laboratory culturing techniques and coculture studies, more network and keystone species analyses, and the expansion of studies to more freshwater iron mat systems are necessary. Increasingly accessible bioinformatic, geochemical, and culturing tools now open avenues to address the questions that we pose herein.

## Abstract (precis)

Freshwater iron mats are dynamic geochemical environments with broad ecological diversity, primarily formed by the iron-oxidizing bacteria. The community features functional groups involved in biogeochemical cycles for iron, sulfur, carbon, and nitrogen.

## INTRODUCTION

The freshwater iron mat environment epitomizes Darwin’s entangled bank (1), with twisted stalks of oxidized iron forming around themselves into charismatic orange mats (2). Iron mats are, as the name implies, comprised of iron oxyhydroxides, the metabolic by-product of iron-oxidizing bacteria (FeOB). They are loosely associated, flocculent structures that can easily be disturbed by an increase in flow. These ephemeral structures also exhibit an oxygen (O_2_) gradient (2), creating myriad niches. While FeOB are diverse in iron oxidation mechanisms (3, 4), the ecology of the microbial communities of freshwater iron mats formed by microaerophilic FeOB is the focus of this review. Previous studies of iron mats have focused primarily on FeOB as ecosystem architects, whereas the literature that focuses on the other organisms in iron mats is sparse (5, 6). Here, we discuss the relationships formed between the microaerophilic FeOB and the other microbial members of iron mats because they drive biogeochemical cycling, ecological relationships, and evolution within these systems. We aim to present the current status of what is known about freshwater iron mat microbial communities and to use this framework to provide direction for future studies.

## ENTANGLED ENVIRONMENTS AND GEOCHEMICAL NICHES

Iron mats formed by microaerophiles have been collected from groundwater seeps, some as cold as 8°C (7), while others have been found in caves (8) or engineered water systems (9). The variability among the freshwater environments where the microaerophilic FeOB exist has been explored in other reviews, and these environments include freshwater environments with FeOB that do not form “mats” (e.g., in the rhizosphere), brackish and marine environments, acidic streams, and engineered systems (9–11). The iron mats that are the focus of this review form in streams where there is a high influx of reduced iron, usually from a groundwater seep, and where the oxic-anoxic interface is near the mat surface, creating both oxic and anoxic microniches within the iron mat (12). Our focus on freshwater iron mats in slow-flow creeks and streams allows us to characterize with some specificity the physical and geochemical environments in which the microbial community forms.

An intricacy of the iron mat environment is that of the physical conditions under which the mat develops. One of these physical conditions is the rate of flow, which has impacts on iron oxidation rates. In studies conducted at Ogilvie Creek, Meilleurs Bay, Ontario, Canada, the presence of an established mat led to higher (1.70 ± 0.20 min^−1^) oxidation kinetics than the ferrous (reduced) iron (Fe^2+^) oxidation kinetics that occurred when the iron mat was artificially washed out (0.48 ± 0.14 min^−1^) (13). This result is perhaps made more interesting by the oxidation kinetics observed for an iron mat formed in a slow-flow drainage channel, which was estimated (0.78 ± 0.20 min^−1^) to be less than half of that of the established mat in Ogilvie Creek, suggesting that oxidation kinetics can be strongly influenced by rate of flow (14). Both studies were conducted in the summer and showed mats dominated by sheaths, indicating that the majority of iron oxidation was carried out by *Leptothrix* spp. It is as yet unknown how a freshwater mat dominated by *Gallionella* spp., or another microaerophilic FeOB, would compare, perhaps leading to variability in oxidation kinetics throughout the year, in keeping with the ecological succession observed by Fleming et al. (15). However, it is likely that a mat dominated by Leptothrix ochracea would have a higher rate of oxidation, considering the rapid production of iron oxides by the species, which is much faster than that of other FeOB (19 μm min^−1^ compared to 2 μm h^−1^) (2). We can draw from this example that the dominant FeOB in the iron mat, as well as the geochemical and physical conditions surrounding the mat, will influence the further ecology within the system.

Consider, for example, the dynamics of dissolved organic carbon (DOC) in iron mats, which have been suggested to correlate with the dominant FeOB taxa in freshwater iron mats, specifically with the occurrence of *Leptothrix*, as opposed to *Gallionella* spp., being closely tied to the presence of higher levels of DOC (15). Because streams are sun exposed, it has been postulated that the presence of DOC may vary due to photobleaching, which would affect the concentration of DOC that is biologically available (15). This is one of many examples of geochemical drivers of iron mat diversity that should be considered and applied to the ecological approach that we aim to present here.

**FIG 1:**
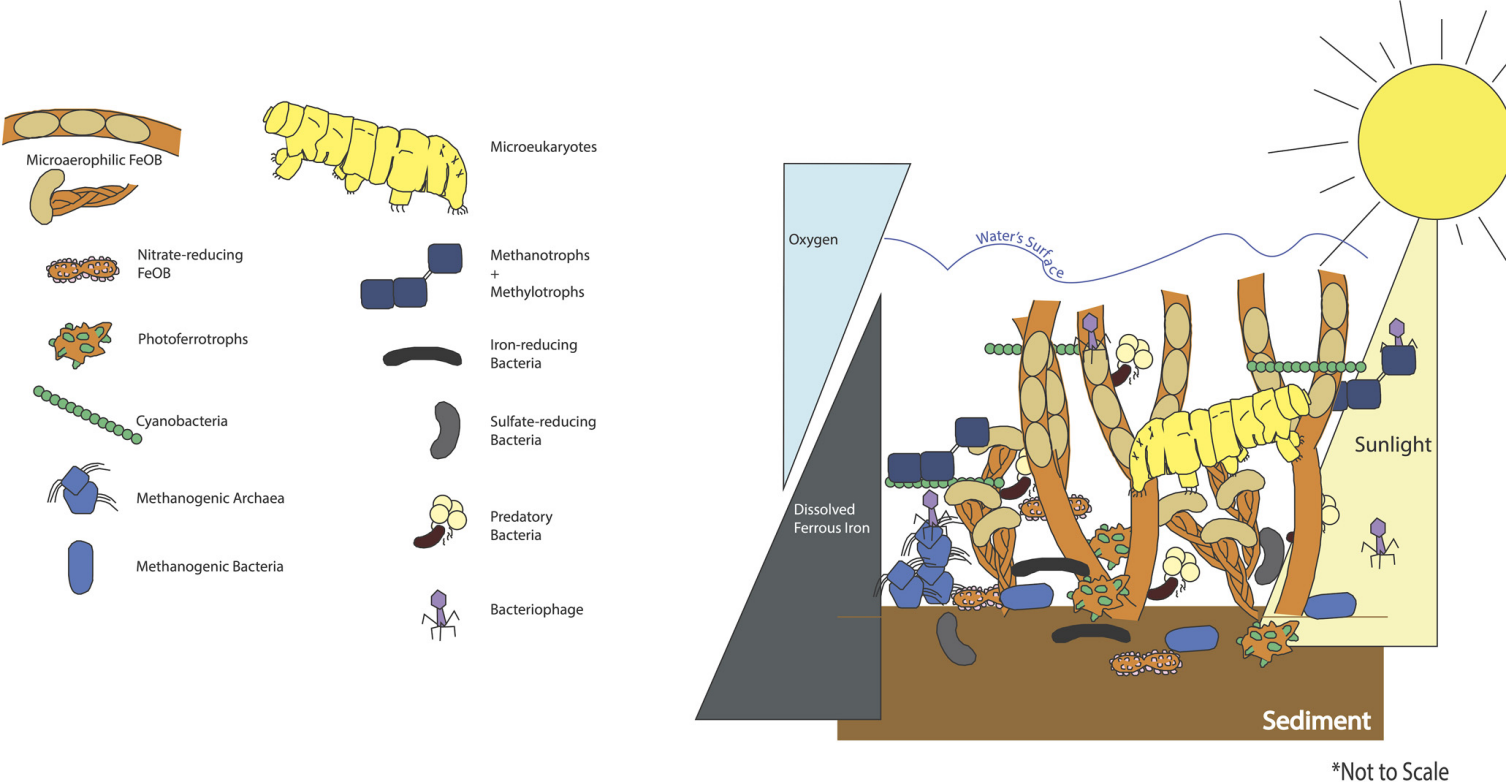
Artistic rendition of some of the notable functional groups present in the neutrophilic freshwater iron mat from eukarya, bacteria, archaea, and viruses. Organisms have been drawn here in their hypothesized niche space based on known functions and abiotic factors, such as sunlight, dissolved oxygen (O_2_), and dissolved ferrous iron (Fe^2+^). Notably, for example, the presence of bacteriophages in the mat and their placement therein are entirely hypothetical, as there is as yet no literature on the niche spaces inhabited by these community members. There are also missing abiotic factors (e.g., organic carbon, nitrogen, phosphorous), which certainly impact the microbial community composition within the iron mat in low-flow streams but are not consistent between mats.

Another example that harkens to a familiar concept in microbial ecology is the presence and biological availability of phosphorous in iron mats. Biogenically produced iron oxides, sometimes referred to as bacteriogenic iron oxides (both use the acronym BIOS) in the literature, have been previously shown to remove phosphorous from solution by adsorption in freshwater as well as other environments, such as marine waters and soils (16–18). Interestingly, there is also evidence that DOC may adsorb to the surfaces of BIOS as well, potentially competing with phosphorous (19, 20) for surface area. While the geochemistry of the iron mat is certainly variable, as shown in the above examples of phosphorous and DOC dynamics, in a freshwater iron mat there are two constants, dissolved oxygen and reduced iron (Fe^2+^), with opposing gradients (Fig. 1). The geochemistry of iron mats certainly impacts the survivability within the stream environment, especially in the formed microniches. As explained here, there may at times be a paucity of biologically available DOC or phosphorous, which may easily lead to shifts in microbial activity and presence.

## IRON MATS: MORE THAN MICROAEROPHILES

The flocculent iron mat often seems to elicit the question of who, or what, lives here? Many functional groups of biogeochemical importance reside within the ochreous confines of the mat (Fig. 1). One functional group that is undeniably present in all neutral, freshwater iron mats is the microaerophilic FeOB. They are keystone taxa, a microbial taxa that exerts a considerable influence on the microbial community structure irrespective of their abundance (21).

The microaerophiles capable of iron oxidation cluster in the class *Betaproteobacteria* and include members of the genera *Gallionella*, *Sideroxydans*, *Ferriphaselus*, and *Leptothrix.* Numerous papers have identified Gallionella ferruginea and Leptothrix ochracea as the primary producers of iron oxyhydroxides in freshwater iron mats using 16S rRNA gene microbial community profiling and characterization of the oxidized iron product (2, 15, 22, 23). *Gallionella* spp. are known to form “stalks,” braided chains of iron at the end of which cells rest, whereas *Leptothrix* spp. produce “sheaths,” tubular iron within which the cells reside (2). Members of the genera *Sideroxydans* and *Ferriphaselus* also produce the stalk structures, which has likely led to some issues of interpretation in earlier studies that used stalks as definitive markers of Gallionella ferruginea’s presence. Interestingly, studies of *Gallionella* and *Leptothrix* spp. have indicated that the two vary in regard to the Fe^2+^ and O_2_ niches that they inhabit, where Leptothrix ochracea has a more flexible response to imperfect gradients (2). This, paired with their apparent dominance in systems with higher concentrations of DOC, has led to the line of inquiry that Leptothrix ochracea may be a mixotroph or heterotroph rather than an autotroph like Gallionella ferruginea (15, 24). While the microaerophilic FeOB are undeniably the stars of the show in freshwater iron mats, there is still more to the story of iron oxidation than that which lies within the micro-oxic region.

Beyond the primary FeOB colonizers, other microbial taxa can be found in the iron mat community. Nitrate-reducing iron-oxidizing bacteria (NRFeOB) functionally exist within the iron mat, though it has been posited that many of these mixotrophic organisms do not actively oxidize iron; instead, they produce a chemical reaction with their metabolic by-products (25). Still, a chemical mechanism of iron oxidation would likely lead to competition between the nitrate-reducing iron-oxidizing bacterial genera *Acidovorax*, *Aquabacterium*, and *Thiobacillus*, which have been identified as present in freshwater neutral iron mats via clone libraries (6, 26, 27). Notably, the nitrate-reducing genera identified from clone libraries were all from the class *Betaproteobacteria*, whereas organisms classified as NRFeOB in other classes were not identified. This is unsurprising, as the average size of clone libraries from iron mats was 97 and *Alphaproteobacteria* made up an average of ∼9% of the clone libraries, when reported (6, 26–30). The other major iron oxidizers, the photoferrotrophs, are also *Alphaproteobacteria* (3). This bias may possibly be due either to selection choices made by experimenters when sampling or to biases that were perpetuated in clone libraries. Regardless, these results indicate that there is perhaps much to be gained from using methodologies that can incorporate greater proportions of the present microbial community.

Today, it is possible to use amplicon sequencing for microbial community profiling, which has aided in the detection of nondominant FeOB and other taxa. Of the current studies that incorporate iron mat 16S rRNA gene environmental sequencing, most did not report the full community profile or mention *Alphaproteobacteria* in their results or discussions (15, 22, 31, 32). Only one reported the incidence of *Alphaproteobacteria*, with an average 9% makeup of Alaskan iron mat communities (22). While this proportion may seem remarkably low, the sample collection for this study was conducted with great care to include only the leading edge of the iron mat, as the authors were interested primarily in the microaerophilic FeOB that are in greater abundance there (22), which likely led to lost data with regard to the presence of members of the *Alphaproteobacteria* that were greater in depth within the iron mat. While appropriate for studies focused on FeOB, experimental designs such as this have likely led to undersampling outside the *Betaproteobacteria* within iron mat communities, potentially leading to biases in our holistic understanding of the iron cycle within the iron mat.

Iron mats feature niches available to organisms other than FeOB, too, which affect where in the iron mat these other organisms are found. Some of the more notable, if understudied, organisms include the predatory bacteria, sulfur-cycling organisms, and methane-cycling organisms (Fig. 1). The predatory bacteria, *Bacteriovorax* spp., have been identified in freshwater iron mats using bacterial clone sequences (4, 6) and likely have a role in maintaining relative abundances in the ecology of the iron mat. Sulfur-oxidizing bacteria (e.g., *Sulfuricurvum* spp.) (6, 27), sulfate-reducing bacteria (e.g., *Desulfobacteraceae*) (29), and methanotrophs (e.g., *Methylophilaceae*) (6) have also been identified using clone libraries. Notably, these include anaerobes and aerobes, possibly competing with the FeOB for niche space or participating in a variety of cryptic nutrient cycles (e.g., carbon, sulfur, nitrogen, phosphorous). It is notably difficult to maintain the structure of an iron mat during sampling, as the flocs are loosely associated and vulnerable to disturbance, and so it is as yet unknowable where exactly in the iron mat each of these organisms would be observed. Here, we present hypotheses based on a general knowledge of the organisms’ oxygen sensitivity, dissolved Fe^2+^ requirements, and photosynthetic capabilities based on the availability of sunlight (Fig. 1). Future studies should aim to maintain the structure of iron mats and study these functional groups *in situ* to tease out their specific niches in the mat.

## WHY ARE MICROBIAL INTERACTIONS IN IRON MATS IMPORTANT?

Microbial relationships are important to the functioning of aquatic environments (33) and biogeochemical cycles (34–37) and in providing colonization resistance against invaders, protecting vulnerable habitats. Microbial communities can be classified using measures of their environmental, functional, and genotypic complexity (38). Using these classifiers for the iron mat community, we can identify knowledge gaps and build a road map for addressing them.

Functional complexity includes considerations of whole-community functions, such as resource use and trade-offs, which create spatial and temporal structural dynamics in microbial communities (39). FeOB alone have been found to be important to the iron cycle (40) via their biological mediation of iron oxidation, which outpaces rates of chemical oxidation in microaerophilic environments (36). However, the functional complexity within the iron mat is reliant upon other microbial guilds, such as the iron-reducing, sulfur-oxidizing, and methanogenic bacteria. How these relationships potentially impact iron cycling has previously been reviewed (41). Because microbial interactions are time-sensitive (42), the variation over time adds another layer of functional complexity to microbial communities, especially those that may have seasonal dynamics (15). Interestingly, many of the functional guilds within the iron mat community are anaerobic, possibly leading to costless metabolic byproducts, so defined as they do not cause a fitness cost to the producer, driving interactions among community members, as this is a trend among anaerobes (43). For example, the iron-reducing bacteria, as a metabolic by-product, produce Fe^2+^, which is then available to other community members or the rapid cycling of sulfate and sulfur by sulfate-reducing and sulfur-oxidizing bacteria, similar to that in the above-described example. Through these machinations, the iron mat community presents a plethora of potentially tied functions and elemental cycles, which in turn makes it a great model not only for microbial ecologists but also for biogeochemists.

Of further importance is the sometimes-cryptic biogeochemical cycling that occurs within these communities. For example, a recent study of freshwater sediment cable bacteria that perform electrogenic sulfide oxidation found that the activity of these organisms enhanced sulfate reduction rates (44). Previously, these effects had not been observed, as this cycling is typically unobservable *in situ*, as they do not lead to an overall increase of sulfate or sulfide concentrations. This example illustrates a commonly observed phenomenon, where the fitness of individuals in a community rely not only on environmental conditions but also on the other members of the population (45). Similarly, there may be many cryptic cycles ongoing in iron mat communities that are not readily observable by traditional chemical measures, such as cycling between FeOB and iron-reducing bacteria (FeRB) or between methanogens and methanotrophs. Using methods of detection, such as 16S rRNA sequencing, is often the only way to hypothesize that such cryptic cycles may be occurring, ultimately leading to experimental setups that may parse out these cryptic relationships.

Genotypic complexity, used here to describe the overall genetic diversity in the microbial community, is the iron mat black box. As DNA yields are often low from iron mat communities, the full genotypic complexity of these communities has rarely been realized. Among the drivers of genotypic complexity are the presence of keystone taxa and keystone guilds (21), such as the FeOB themselves, which are responsible for niche partitioning (4). Iron mats create niche spaces available to other functional guilds due to the opposing gradients of oxygen and reduced iron, setting the stage for the relationships that we will discuss here. According to a study of seasonal changes along a freshwater first-order stream in Boothbay Harbor, ME, the keystone taxa within the FeOB changes temporally, with the dominant iron oxidizers shifting from *Gallionella* spp. early in the year (April) to *Leptothrix* spp. in the summer (June) (15). This specific trend may not hold true for all iron mat communities, especially iron mat communities in geographical locations not affected by snowpack and subsequent snow melt, which impacts O_2_ dissolution in the water column. However, common to all iron mats, beyond the opposing Fe^2+^ and O_2_ gradients, are environmental factors such as wastewater runoff, nutrient loading, and flow; these factors are all often variable in the urban environments where many mats are located. How these factors may, independent of season, impact the dominant FeOB and, perhaps subsequently, the colonization by other functional guilds is as yet unclear.

Each of these classifiers of complexity (environmental, functional, and genotypic) in the community can affect the others. For instance, as the global climate changes, the microbial diversity in many types of communities has experienced shifts in response (46). This change in the environmental complexity, where typical conditions are no longer typical, has led to shifts in the observed functional and genotypic complexity. Ostensibly, this changes the rates of mortality within the communities that are sensitive to the removal of keystone species, colonization by invasive species, and global climate change (47). The iron mat community may be more impervious to the effects of global climate change than many other microbial communities given that the FeOB appear to be adapted to temperate conditions, as in the study in Boothbay Harbor, ME, where the mats are not present in the winter (15); however, the freshwater communities associated with iron mats may still be at risk. As mentioned previously, one of the possible drivers of available DOC in streams with iron mats is photobleaching. This particular condition can be attenuated with an increase or decrease in rainfall, which correlates with an increased or decreased albedo, respectively, changing the rate at which DOC is photobleached. Changing weather patterns may also lead to saltwater intrusion in iron mat sites that are upstream of estuaries, one example being the freshwater mat upstream of brackish waters in the Sheepscot River, ME, study (23). Sites such as these are vulnerable to increased intrusion due to drought and sea level rise. Changes on a global scale can certainly have local-scale effects that even the freshwater iron mat may experience, leading to shifts in the microbial make-up and function of these ecosystems.

## SYNTROPHY: COMMUNITY ASSEMBLY, STRUCTURE, AND FUNCTION

The study of syntrophic relationships between microbes in iron mat communities lies primarily in theory (48), but many important findings from synthetic microbial communities can be applied toward the study of *in situ* microbial communities, such as that of the iron mat. For example, two cocultured organisms, *Xanthomonas retroflexus* and Paenibacillus amylolyticus, developed phenotypes that enhanced their ability to grow in a biofilm together (49). It is likely that similar adaptations, i.e., the bolstering of survival traits, may occur in natural environments, including the iron mat. It is, however, more tractable to study how the cooccurrence and cooperation between microbial groups may drive community structure of established communities (50).

Cooperation is an important driver of community function, especially under environmental stress. It has been observed that generalists, when facing lost advantage due to perturbation, will increase syntrophic processes (51). Syntrophic relationships can also be important for the function of microbial communities in carrying out biodegradation pathways. Using stable isotope probing, syntrophic relationships leading to the removal of hydrocarbons have been identified between iron-reducing bacteria and sulfate-reducing bacteria (SRB), as well as methanogens and acetate oxidizers (52–56). These relationships are of particular interest, as they involve functional groups present in the iron mat system. Such cooperative relationships between microbes may have global import in the form of connecting biogeochemical cycles, potentially extending to many of the Earth’s biogenically controlled cycles (57), including sulfur (7, 23, 58), nitrogen (8, 30), manganese (59), and carbon (5, 6, 8).

Syntrophic relationships between the marine FeOB and their community members have been explored to greater depth than the relationships in the freshwater iron mat have. Still, potential syntrophies have been postulated between the FeOB and cooccurring functional groups, including SRB (7, 23) and oxygenic phototrophs (60). The potential for connections extends outside FeOB; SRB and methanogens are well known for their syntrophic capabilities (61–63). The methanogenic microbes involved in these syntrophic interactions are reliant on other functional groups for electron donors, and their syntrophs are typically H_2_ or formate scavengers that can switch to a sulfate reduction pathway, where they may begin competing for acetate, depending on the carbon-to-sulfate ratios. Methanogens in anoxic cultures from a rice paddy field have also been observed to build syntrophic interactions with FeRB that are facilitated by iron oxide particles (64). The results of the study suggest that *Geobacter* spp. benefit from increased growth, and the methanogen *Methanosarcina* spp. was able to increase the rate of methanogenesis via an electromethanogenesis pathway (64). Microbial syntrophies in the iron mat likely play a large role in modulating the growth rate of organisms *in situ* and studies designed to capture this would strongly contribute to the literature.

**FIG 2:**
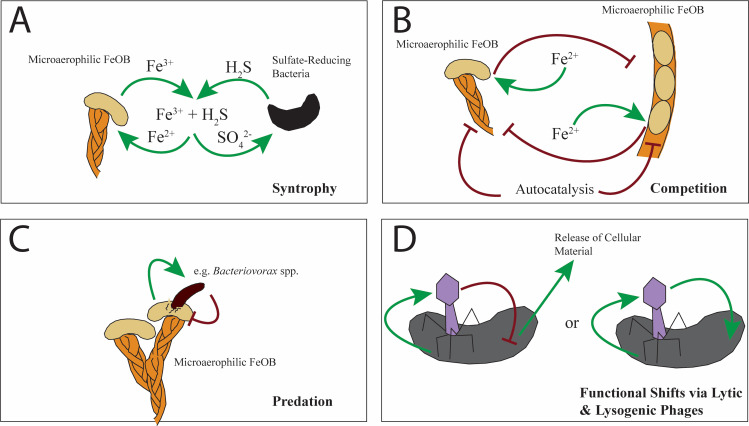
Brief graphical summary of some of the potential relationships that may work to maintain the iron mat community. (A) Syntrophic relationships have been proposed for functional groups that coexist within iron mat communities, for example, the potential relationship between microaerophilic FeOB (e.g., *Gallionella* spp., *Siderooxydans* spp., *Ferriphaselus* spp., or *Leptothrix* spp.) and sulfate-reducing bacteria that have been identified in freshwater iron mats via 16S rRNA sequencing (7, 23). (B) Competitions for niche space and resources is likely prevalent in the iron mat community, though how this competition impacts growth rate is currently unknown. Here, the competition is between two different microaerophilic FeOB competing for Fe^2+^ within their shared niche space; this competition is also augmented by the formation of Fe^3+^ chemically, known as autocatalysis, the rate of which has been previously investigated (117). (C) Predation within iron mat communities, particularly that of bacterivorous species, such as *Bacteriovorax* spp., has not previously been considered as having a large impact; however, rates of predation may influence dominant taxa or the ecosystem architects, the Gram-negative FeOB. (D) Two of the possible interactions between bacteriophages and their bacterial hosts, either as antagonists (e.g., cell lysis) or as symbionts (e.g., metabolic regulation), that have been shown to modify local ecology. The study of bacteriophages within iron mats is a field as yet unexplored.

Perhaps of greatest interest are the syntrophic relationships that may form between the ecosystem architects and the community members. There are certainly well-known examples of this, such as the syntrophy between FeOB and the iron-reducing bacteria, reviewed elsewhere (65), but there are other, perhaps overlooked, possibilities that we wish to present here. The syntrophy between FeOB and SRB, where the cooccurrence is well established in the marine system, is likely mediated by the O_2_-Fe-H_2_S catalytic cycle (66–68), where reduced iron and sulfate are produced from the reaction of oxidized iron and hydrogen sulfide, making the microbial waste (oxidized iron and hydrogen sulfide) back into microbial food (reduced iron and sulfate) (Fig. 2A). The most practical implication of this relationship is that the iron mat’s chemistry may feasibly sustain both FeOB and SRB during times of low availability of either reduced iron or sulfate. While first observed in marine systems, the cooccurrence of FeOB and SRB has been noted in freshwater systems as well (7, 23) and could be of great importance during the establishment of iron mats, where the sediment community likely serves as a microbial seedbank (23). This may potentially expand the range of environmental conditions where iron mats can be formed and may add further stability to the iron mat microbial community composition. Novel coculture conditions have been recommended for marine FeOB and SRB (69), which may be applied to freshwater guilds, but additional cultivation methods may be warranted for future growth-based studies of these two guilds in controlled laboratory settings. While freshwater and marine FeOB communities are disparate with regard to physical, chemical, and biological characters, it may still be informative to draw upon the marine community for functional ideas; as this example shows, there is much functional overlap between the two.

Of course, there are other potential syntrophies with FeOB that merit further investigation. FeOB may also form a syntrophic relationship with planktonic cyanobacteria in the freshwater iron mats. While this has not been explored in freshwater iron mats, it has been suggested under brackish conditions (60). In this instance, the cyanobacteria may be protected from oxidative stress due to the presence of reduced iron species, while the FeOB receive localized O_2_ produced by the phototrophs when bulk water O_2_ concentrations are too low (60). However, as with any syntrophic relationship, it is possible that this alliance may change in nature under different conditions. In this case, it has also been observed that the growth of acidophilic FeOB in iron mats has been stymied by the presence of cyanobacteria (70). This dynamic is likely due to the degassing of O_2_ from acid mine drainage, leading to an increased organic carbon-to-O_2_ ratio from the presence of photosynthetic organisms, which ultimately leads to greater competition between the FeOB and organisms bolstered by the increased organic carbon (70). In a neutrophilic freshwater iron mat, it is likely that the increased O_2_ from the presence of phototrophic organisms would be of greater benefit, as with the brackish conditions previously mentioned. This example demonstrates that not only marine, but acidophilic, iron mats may be useful in hypothesis generation. However, the ultimate test of these syntrophic relationships will come from further study in the freshwater iron mat system itself.

## COMPETITION AND PREDATION: NICHE PARTITIONING AND COMMUNITY COMPOSITION

Competition and predation, much like syntrophic relationships, are difficult to study *in situ*; however, these questions are arguably more tractable in a simplified community, such as those in a freshwater iron mat, given the complexity in, for example, soil systems. It has been noted that competition can increase microbial diversity by competitive exclusion and negative frequency-dependent selection (71). Similar controls are exerted by predation; in a controlled experiment, it was observed that some typically rare taxa (e.g., *Comamonadaceae*) in a model bacterial community had the highest abundance when the protistan predators were removed (72). It has also been suggested that functional redundancy is, at least in part, maintained by competition and predation (73). These observations may have interesting implications for the interpretation of relative abundance, often used to reconstruct community structure, in freshwater iron mat communities.

One of the most obvious competitions in iron mat communities is that between the microaerophilic FeOB themselves. Those most often studied are Leptothrix ochracea and *Gallionella* spp. While these organisms have been shown to coexist in some iron mats (2, 28), they have also been shown to have an almost mutual exclusivity based on current environmental conditions (15), indicating that these organisms share the same niche space and may be competing at the microscopic level (Fig. 2B). However, it is easily forgotten that in these same freshwater environments, there are other organisms competing for reduced iron, namely, the photoferrotrophs (37, 74–76) and the NRFeOB.

Competition among microbial taxa that utilize the same resources is likely to occur in freshwater iron mats. In a study of coastal iron cycling communities in near-shore marine environments of Aarhus Bay, Denmark, Laufer et al. observed microaerophilic, nitrate-reducing, and phototrophic FeOB coexisting in two different sediment types (77). In a stark difference from what has been observed in a study of iron mats (5), the sediment communities of FeOB observed were not stratified according to O_2_, Fe^2+^, or light conditions (77). The authors postulate that this was due to physical turbulence and bioturbation in the marine sediments, which would be less effectual on a typical iron mat. However, this study suggests that the shared niche spaces of the three types of iron oxidizers in freshwater iron mats, where low-flow streams are less turbulent, may lead to heretofore-unobserved competition between the groups; certainly more studies are warranted.

Other functional groups, the methanotrophs and methylotrophs (5, 6, 8), may also compete with the microaerophilic FeOB for the available oxygen in the iron mats (5). Quaiser et al. found methane-oxidizing bacteria to be a significant proportion of the iron mat microbial communities (5), suggesting that this competition may be widespread and drive oxygen cycling in the mat. This interaction has not been well studied, and the notable organisms have likely been undersampled in clone libraries, given that they are not *Betaproteobacteria*.

The role of predation in altering the biogeochemical potential of the microbial community is likely large, but as yet, no studies of predation in the iron mats have been conducted. Notably, *Bacteriovorax* spp. have been identified in iron mat communities (4, 6) and are known to prey on Gram-negative bacteria (78), possibly shaping the iron mat community (whose architects, the microaerophilic FeOB, are notably Gram negative) (Fig. 2C). Predation by bacterivorous species is typically indiscriminate and has been found to significantly alter relative community compositions (79, 80). This may have important implications for any applied uses of iron mat communities, especially in the transfer of iron mat seed banks to novel locations with higher or lower bacterivorous species incidences than *in situ*.

## EUKARYOTES, VIRUSES, AND ARCHAEA, OH, MY?

What roles do microeukaryotes, viruses, and archaea play in iron mat microbial communities? The other branches of life are not only largely missing from the iron mat literature, they have often been overlooked in studies of all environments (81, 82). Microeukaryotes and archaeal iron mat constituents rarely appear in the literature (5, 6). One study identified nine archaeal phylotypes (6), and another reported sequencing two archaeal transcripts (5). Microeukaryotes identified from iron mat transcripts were associated mostly with freshwater grazing species (e.g., *Tetrahymena* spp.) (5), which have previously been observed to have a role in increasing bacteriophage and bacterial encounters by accumulating both in their phagocytotic vesicles (83). Clearly, the role of microbes other than bacteria in the iron mat should not be brushed off as ancillary. Microeukaryotes have also been shown to modify the community structure and abundances in bacterial communities, as predation can lead to a rarity of fast-multiplying bacterial taxa *in situ* (72). This predation by microeukaryotes may be especially relevant to iron mat communities, where one of the keystone taxa, Leptothrix ochracea, has a rapid doubling time of 5.7 h (24), which may lead this organism to be underrepresented in community sequences. Rare bacterial species in an environment may have invested less in defenses against grazing with bacterial phenotypes such as cell size and cell wall structure (84) and instead may have invested more in quick replication (72). This response to predation can also lead microbial communities to upregulate bioremediation processes (85), which may prove an essential element to the application of iron mat communities to polluted environments. Microeukaryotes, it should be noted, do not parody bacterial community members in community structure shifts. While there can be temporal structure and functional change (86), microeukaryotes are more likely to respond to deterministic processes in marine ecosystems, unlike bacteria and archaea, which appear to respond more strongly to stochastic processes (87). This trend has been hypothesized to be driven by strong adaptation capabilities in prokaryotes; alternatively, environmental factors that have the most relevant impact on prokaryotic community members are not being measured (87). In studies of iron mats, it may be of use to use microeukaryotes as “canaries in the coal mine” to identify the relative stress (i.e., deterministic processes) that the community is facing. For example, facing ecological severity from the Deepwater Horizon oil spill, microbial communities increased in bacterial dominance over archaea and microeukaryotes (88). The role of microeukaryotes in the freshwater iron mat is largely unexplored, but the datum that is available points to ecologically relevant roles within the ecosystem.

Returning to the prokaryotic organisms among the iron mat, there is also a scarcity of information on the archaea present in freshwater systems. It is not clear what role the archaea may play in the iron mats, as they currently represent a very small proportion of available iron mat community sequences (5, 6, 31, 32), often being identified secondarily only through the use of bacterial primer sets. As this does not encompass the majority of the archaeal diversity in the environment and likely in the iron mat, we conducted Illumina MiSeq sequencing of seven freshwater iron mats from Greenville, NC, using the archaeal primers A956F (TYAATYGGANTCAACRCC) and A1401R (CRGTGWGTRCAAGGRGCA) (89). Sequences were processed using mothur (v 1.44.1) (90–92), and the MiSeq SOP was accessed 13 April 2020 (https://mothur.org/wiki/miseq_sop/) to identify present taxa (97% operational taxonomic unit [OTU] threshold). Graphs were generated using the phyloseq package (93) in R v3.5.2.

**FIG 3:**
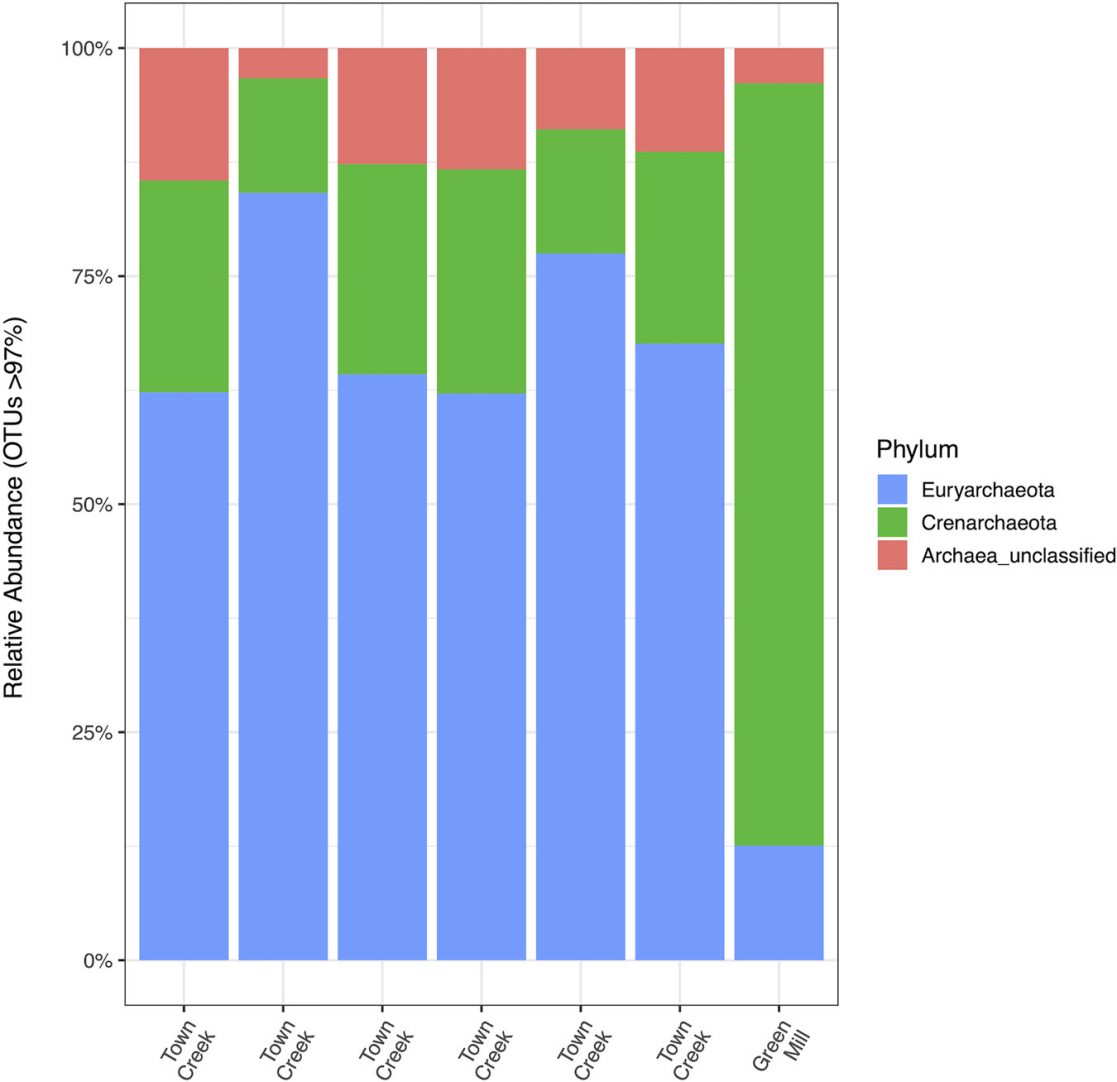
Archaeal 16S rRNA genes were sequenced from seven urban-area-impacted freshwater iron mats in Greenville, NC. Six of the iron mats were sampled from Town Creek, and an outgroup from Green Mill Run was included. The relative abundances of the phyla are represented here. *Euryarchaeota* (blue) account for 43%, *Crenarchaeota* (green) account for 24%, and unclassified *Archaea* (red) account for 33% of the total archaeal sequences from all seven iron mats.

Through the use of a targeted archaeal primer set, we were able to amplify a much higher abundance and diversity of archaeal amplicon sequences than the proportions previously reported. Among all seven of the iron mat communities included in this analysis, there were 1,699 total archaeal OTUs identified, with an average of 400 archaeal OTUs per mat, demonstrating that the archaeal diversity is higher than previously shown. The most abundant phylum was *Euryarchaeota* (Fig. 3), which accounted for 43% of the total archaeal sequences. Eleven percent and 1% were *Methanomicrobiales* and *Methanobacteriales*, respectively. Sequences of these methanogenic archaea were found in all seven iron mats, suggesting that their widespread presence in the iron mats may be important for the biogeochemical function of the iron mat community as a whole and that further efforts should be made to recover more complete sequences of archaeal community members from more diverse iron mats. Furthermore, cultivation and cocultivation techniques should be employed to further delve into the interactions between archaea and bacteria in the iron mat.

Another area of study ripe for investigation is the role of bacteriophages in the iron mat community. Viruses impact microbial communities through varied mechanisms, with effects such as community turnover (94) and changing bacterial abundance and function (95). Archaea and bacteria can also benefit from lateral gene transfer between themselves, and this benefit can be mediated by viruses (94). Functional shifts can occur due to the presence of auxiliary metabolic genes present in both lytic and lysogenic phages (Fig. 2D). These genes have been observed to modify host dynamics in marine systems, with auxiliary metabolic genes modifying host metabolic needs or redirecting all cellular energy toward phage replication; further details of these mechanisms have been reviewed by Warwick-Dugdale et al. (96). As with microeukaryotes, viruses result in top-down pressure in bacterial communities (97). Even a community low in viral diversity can experience a large impact from viruses, given the variability in host specificity (98). Viral community members may also help to maintain and shape communities, even while in a steady state (84). Interestingly, in the first temporal study of riverine viromes, conducted in three watersheds in British Columbia, Canada, the viral communities were distinct between sites, even those where the geographic distance was markedly close enough for the bacterial communities to be similar (99). Notably, this study also found that the communities of both DNA and RNA viruses were synchronous (99), possibly owing to more similar environmental conditions impacting viral community members that are not analogous in effect to bacterial community members. As yet there have been no similar studies conducted in iron mats, but in seeking data from a related environment, in this case a river, we have aimed to show the possibility for hypothesis generation from these data sets to be applied to the iron mat system.

## THE SOLUTION TO POLLUTION IS…IRON MATS?

Iron oxyhydroxides produced by FeOB have been studied for their abilities to combat anthropogenic pollution by leaching heavy metals (20, 100–102), degrading aromatic carbons (8), adsorbing hydrophilic pesticides (103), and removing phosphorus (16, 104, 105) from contaminated waters. The iron mat microbial community has a diverse ability to degrade and transform these contaminants, ultimately affecting their fate, but the presence of these contaminants will also be a stressor to the community itself and its functioning. The iron oxides are known to remove phosphorous from solution and the biologically available pool through sorption mechanisms (16). Because of this, biologically produced iron oxides have also been applied in remediation strategies, where they similarly adsorb arsenic (106). However, few studies have addressed the entire community involved, not only those bacteria identified as responsible for contaminant degradation. By expanding studies to include a more holistic view of the entire community (e.g., bacteria, eukaryotes, viruses, archaea) in the iron mat, we can better understand how their complex interactions affect community functions, such as contaminant degradation and transformation. For example, heavy metals and hydrocarbons can induce the formation of reactive oxygen species, which are toxic to bacterial species (107), potentially leading to changes in the overall microbial community in the affected iron mat. Responses of microbial communities to anthropogenic stressors are dynamic (108) and highly context dependent (107). The responses of microbial communities depend on the pollutant, whether it be heavy metals, which often lead to decreases in diversity (109, 110), or polycyclic aromatic hydrocarbons (PAH), where communities may decrease (111, 112) or recover diversity after chronic stress (113, 114).

Again, we see the importance of geochemical factors in the regulation of microbial communities when we consider pollution. In the Yangtze Estuary in China, both PAH and heavy metals are contaminating the estuarine sediment. Importantly, not only were the PAH and heavy metals responsible for regulating the degradation potential of the microbial community, but pH and salinity also played a role (107). Environmental severity, as defined not only by the concentration of pollutants but the surrounding environmental factors, plays a role in the degradation potential of the microbial communities. Key to this study was that the microbes harvested naturally occurred in the polluted area, and still, the environmental factors outside of pollution had significant effects on the degradation potential (107). The functional groups of the iron mat are commonly thought of as sensitive to oxidative-reductive potential (ORP), dissolved O_2_, and physical factors (e.g., flow); how these niche-defining environmental cues interplay with contaminant presence in the iron mat to impact the microbial community is an exciting new avenue for future research.

In urban environments, the presence of all of these contaminants in the same iron mat would come as no great surprise, easily increasing the environmental pressure experienced by the microbial communities of the iron mat. A focus exclusively on the degradation potentials of these mats can obscure the importance of these stressors on ecological networks in the iron mats and the role of keystone species. In a study of riverine sediments from Suzhou, China, that were contaminated with hydrocarbons, the keystone bacteria (e.g., *Dechloromonas* and *Anaerolineaceae* spp.) were able to facilitate interactions, even as the concentration of hydrocarbons increased (115), supporting the biodegradation of contaminants. As the hydrocarbon concentrations increased, the strength of the species aggregations increased as measured using the Molecular Ecological Network Analysis Pipeline, indicating a greater importance of keystone species to environmental function (115).

Excitingly, functional groups found in the iron mat appear to have potential in the removal of contaminants from waterways. In a study using isolated FeOB and SRB from sewage sludge of Xiangtan City, China, cocultures were more effective at attenuating antimony [Sb(V)] than isolates (116), indicating the importance of these interactions in contaminant transformations and community function. Similar mechanisms likely play out in iron mats, which are often found in urban environments, such as the North Carolina Piedmont (20), that are prone to increased pollutants. Studies of these and other urban iron mats may lead to the potential application of the holistic microbial communities, not only the bacteria, toward the attenuation of PAH, heavy metals, or other contaminants. Future avenues of research include using -omics techniques, *in situ* observations, and culturing techniques to understand how microbial interactions in the iron mat relate to contaminant remediation.

## CONCLUDING REMARKS

Community sequencing, of both 16S rRNA genes and metagenomes, can be leveraged to understand the taxonomic and functional diversity within the iron mat. This may be particularly useful where we do not yet have geochemical data and cryptic biogeochemical cycles may occur. While we have a strong foundation of knowledge of the role of iron-oxidizing bacteria in the iron mats, there is still much to be garnered from current and future data sets to expand sequencing and studies beyond these bacterial members to incorporate other functional guilds and microeukaryotic, archaeal, and viral members’ roles. We also hope to see an inclusion of network ecology approaches, studies of indicator species, and the development of novel coculture techniques toward discovering and understanding specific interactions within the iron mat community. Applying these approaches may reveal much-needed information about other key taxa in iron mat communities, perhaps also revealing some of the more cryptic relationships and functional roles of these iron mat communities, such as contaminant degradation in these environments. Many research directions remain in the field of iron mat microbial communities, including exploring viral and eukaryotic communities, competition and predation, syntrophic relationships, and the impacts of anthropogenic stressors. While the iron mat is host to a great diversity, it is also simple in comparison to many other freshwater communities and provides an accessible model system for testing ecological theories and interactions between the domains. Here, we recommend that researchers strike while the iron is hot and work toward building a greater knowledge base for this exciting community.
